# Memory difficulties in perimenopause: neuropsychological distinction from early-onset dementia

**DOI:** 10.1192/j.eurpsy.2026.11940

**Published:** 2026-07-31

**Authors:** L. G. Nunes

**Affiliations:** Mackenzie University, São Paulo, Brazil

## Abstract

**Introduction:**

Memory complaints in middle-aged women are often misinterpreted as signs of mild cognitive impairment (MCI) or early-onset dementia. However, perimenopause represents a period of neurobiological vulnerability associated with estrogen decline and transient hypometabolism in the hippocampus and prefrontal cortex. This case highlights neuropsychological parameters useful for distinguishing functional cognitive changes from amnestic-type neurodegenerative syndromes.

**Objectives:**

To highlight how neuropsychological assessment can distinguish functional memory impairment related to perimenopausal hormonal changes from early-onset dementia in women with high cognitive reserve.

**Methods:**

A 49-year-old female judge, with a Master’s degree in Law, no psychiatric history, under hormonal replacement therapy and GLP-1 agonist use, reported progressive forgetfulness over six months. A comprehensive neuropsychological assessment was conducted, including WAIS-IV, RAVLT, FDT, and BPA.

**Results:**

Full-Scale IQ = 120 (+1.3 SD). Verbal Comprehension ≈ +1 SD, Perceptual Organization ≈ +0.7 SD, Processing Speed ≈ +0.8 SD.

RAVLT revealed a **progressive learning curve** with **marked loss after interference** and **limited recognition gains**, indicating a weakness in **consolidation (−1.3 SD delayed recall)** rather than attentional control.

Immediate recall was average (0 SD), delayed recall low-average (−1.3 SD).

FDT showed **reduced cognitive flexibility (−1.3 SD)** with preserved inhibitory control.

BPA indicated attention performance ranging from **average to low-average** (alternating ≈ −0.3 SD, divided ≈ −0.6 SD, sustained ≈ −1.0 SD).

**Conclusions:**

The pattern reveals high intellectual functioning with isolated reduction in long-term verbal memory and cognitive flexibility. The learning curve profile and post-interference decay indicate **functional hippocampal hypofunction** linked to hormonal and metabolic changes of perimenopause, rather than neurodegeneration.

**Disclosure of Interest:**

None Declared

